# Do We Ask What the Deities Can Do for Us? The Roles of Dao Religion and Resilience in Suicidality in Chronic Pain

**DOI:** 10.1155/prm/3056383

**Published:** 2025-04-17

**Authors:** Ling-Jun Liu, Hsiu-Ling Peng, Edward Meng-Hua Lin, Wan-Ping Liang

**Affiliations:** ^1^Department of Anesthesiology, Changhua Christian Hospital, Changhua City, Taiwan; ^2^Department of Statistics, Tunghai University, Taichung City, Taiwan; ^3^Department of Psychology, Chung Shan Medical University, Taichung City, Taiwan; ^4^Department of Pastoral Care, Changhua Christian Hospital, Changhua City, Taiwan

**Keywords:** chronic pain, mixed-method study, religiosity, resilience, suicide

## Abstract

**Objectives:** Resilience to pain is a protective factor against aversive pain outcomes, such as suicide. Religiosity as a cornerstone of resilience has been found to be associated with reduced risk of suicidality in chronic pain. However, affiliations to different religions have displayed differences in suicide risk. This study focuses on the roles of pain resilience and Dao religion in mitigating suicidal experience in individuals with chronic pain.

**Methods:** This study adopted a mixed-method approach. A preliminary investigation was conducted regarding the internal consistency and construct validity of the translated version of the pain resilience scale (PRS). Qualitative data were collected through interviews with individuals experiencing chronic pain. Levels of PRS and gender were included in the logistic regression on the probability of suicide attempts. The role of Dao practice was qualitatively analyzed through narrative analysis.

**Results:** Among the 24 participants, 14 were affiliated with the Dao religion; therefore, the transcripts of these 14 interviews were analyzed. Individuals with moderate scores on the PRS were 11.60 times less likely to have attempted suicide than those with low PRS scores. The likelihood further decreased by 38.7 times in those with high PRS scores. Four themes emerged from the qualitative interviews. The participants experienced a burden from pain, made efforts to please the deities in exchange for better pain control, continuously adjusted to pain, and ultimately developed a new perspective on the relationship between their religion and pain. Many individuals have engaged in Dao rituals to try to alleviate their physical and psychological pain. Most participants tended to offer a religious interpretation of enlightening moments after surviving a suicide attempt.

**Discussion:** This study illustrates how pain resilience and Dao religious practices mitigate suicidality in chronic pain.

**Trial Registration:** ClinicalTrials.gov identifier: NCT05148364.

## 1. Introduction

Resilience for pain may refer to various forms of adaptations to chronic pain and the resources supporting this adaptation [1]. It can be defined as the maintenance of a healthy lifestyle [2], remission of physical symptoms [3–5], psychological positivity [6–8], and social resources that help cope with pain [2]. This resilience for pain can lead to different pain outcomes [1]. Most research regarded resilience as a protective characteristic against aversive pain outcomes. With a lack of resilience, the worst possible outcome of pain management may be ending one's life [9, 10].

Religiosity and spirituality are two important components of resilience [11–13], and they have been recognized as protective factors for suicide prevention [14–16]. In a systematic review that included 41 studies, the authors described many common factors underlying religiosity/spirituality and resilience; however, they should be considered independent concepts [12]. Healthy religiosity/spirituality is associated with increased resilience, but the reverse was not always true [17]. Religiosity itself was found to be a highly effective predictor of resilience [18]. In addition to the moral objections against suicide, certain positive factors in religious practices can potentially protect an individual from ending one's life. Scholars have identified connectedness as a protective factor against suicide [19–21]. Many of the previous studies on this topic were conducted in a predominantly Christian society. According to a questionnaire-based study that included approximately ninety thousand participants, mainly Protestants and Catholics, the frequency of religious attendance was found to be negatively associated with the rate of suicidality [22]. In another study, church attendance was associated with a lower risk of suicide ideation, and this relationship was partially determined by perceived social support [23]. Even for individuals with pain, whose risk of suicide increased due to the illness [16, 19, 24, 25], a higher level of spiritual well-being was associated with a decreased level of pain catastrophizing [26].

Religious individuals are more likely to engage in positive coping strategies than nonreligious individuals [27]. Religious practices, such as seeking a spiritual connection, using religion as a distraction, asking for forgiveness, and finding solutions with God, can serve as a mechanism for pain tolerance and better psychological functioning [12, 28, 29]. Individuals with chronic pain who regard religion as a salient part of coping are more likely to report feeling supported and present better mental health [30]. Generally, religiosity may have a protective effect against negative mental health outcomes, such as depression or suicide, when dealing with chronic illness [31, 32]. It is also linked to remission from suicidal ideation in chronic pain [33]. However, affiliations to different religions have indicated differences in suicide risk [34, 35].

Many reviews have examined the association between suicide and major religions such as Christian, Hinduism, Islam, and Judaism, but there is less research on Dao and suicide [36]. A study on religious belief and suicidality in China found that the correlation between the two was not a straightforward linear correlation. That study included participants from different religious backgrounds: Christianity, Buddhism, Islamism, Daoism, and Catholicism [37], and its results indicated that the relationship between suicidality and religious belief was moderated by the level of political belief. Another questionnaire-based study conducted in Taiwan included participants from four predominant religions in the region: Christianity, Buddhism, Daoism, and Catholicism [34]. In that study, Christian reported the highest rates of suicide ideation and suicide attempts, while the other three groups showed no significant differences. Unfortunately, neither study collected narrative data on how the participants from different religious affiliations experienced suicide attempts or how religion influenced their recovery from a suicide attempt (i.e., resilience).

Contemporary Chinese culture is strongly influenced by Daoism, Confucianism, and Buddhism [38]. Several studies have examined how individuals with a Chinese cultural background deal with pain [39, 40]. However, few studies have clarified that Daoism is distinct from the Dao religion. Dao was one of the philosophical schools of thought that originated in the Warring States period (475–221 BCE) before the Han dynasty [41]. Dao means “the way” and refers to the course and order of the universe [42]. It encompasses concepts, such as Yin–Yang (陰陽) and Wuxing (五行), that refer to the ways in which the universe operates. According to Daoism, Dao is fundamental to human society.

Although prophylactic treatments include certain aspects of Daoism, such as Qigong and Yin–Yang in Chinese medicine [43–45], the most common practice in daily life is the Dao religion. Dao religion was derived from Daoism during the late Han dynasty [41]. Dao as a religious practice, distinct from Dao philosophy, incorporates mystical figures as objects of worship [46], rituals, and doctrines that dictate human behavior in this religious cultural context [47]. In addition, many practices of the Dao religion share similarities with those of Buddhism [48].

Previous studies have attempted to delineate how individuals interpret chronic pain from the philosophical Dao perspective [39]. However, research on how Dao religious practices are implemented in day-to-day chronic pain management is scarce. Research on Dao religion and suicidality is limited, and even fewer studies have explored the relationship between Dao religion and physical pain. Since the present study was conducted in an area where Dao religion is the most common religion, it should focus on participants' experiences that align with findings from other religions as well as those that are unique to Dao. According to open data from the Ministry of the Interior of Taiwan, more than 78% of religious facilities are registered as Daoist-affiliated [49]. This study examines the role of Dao religion in individual's suicidal attempts, particularly in relation to resilience against pain. There appears to be a need for further investigation. To the best of our knowledge, no studies have discussed the role of Dao religion in mitigating suicidality in individuals with pain. Given the lack of research on the relationship between Dao religion, resilience, and suicidality in patients with chronic pain, there is a potential for further investigation. Therefore, this study aimed to investigate individuals' interpretation of chronic pain in the context of the Dao religion and resilience fostered by Dao religion practices in managing pain-related suicide attempts.

## 2. Methods

This study adopted a mixed-method approach to incorporate theory-based parametric variables with the subjectivity of individuals with chronic pain. As mentioned in the introduction, studies on Dao religion and suicidality are scarce, and many previous studies on religion and suicidality have been questionnaire-based, limiting variables and the scope of responses to the predefined questions. The addition of qualitative interviews in a mixed-method approach not only allowed for a more substantive exploration of the topic of interest [50, 51] but also helped capture the complexity of individual experiences on this seldom-discussed topic. A brief overview of this mixed-method study: To investigate the roles of pain resilience and Dao religion in mitigating suicidality among patients with chronic pain, we first validated the pain resilience scale (PRS) and used it to assess the pain resilience of the interviewees. Qualitative data provided deeper insights into the topic of interest. Finally, the findings were verified through statistical analysis.

The study conducted a preliminary investigation on the reliability of pain-resilience measurement. It was approved by the Internal Review Board of Changhua Christian Hospital (approval number: 181230, 201117) and the Internal Review Board of Chung Shan Medical University Hospital (approval number: CS2-20186).

The main theme of the present study comprised interviews with the participants, and this part was approved by the Internal Review Board of Changhua Christian Hospital (approval number: 211102). Preregistration information is disclosed on the title page. Participants were recruited from a pain clinic at a medical center, Changhua Christian Hospital, in central Taiwan. It is a large healthcare and academic facility with more than 5000 healthcare professionals and 1400 primary care wards. It offers medical trainings, research, specialty clinics, emergency treatment, tertiary care, and surgery care. Pain clinic is one of the specialty clinics in this medical center, primarily serving patients with chronic noncancer pain and some patients with cancer-related pain. Written informed consent was obtained from each participant prior to the interviews.

### 2.1. Transparency and Openness

We report how we determined our sample size and all data exclusions, manipulations, and measures in the study; we follow the Journal Article Reporting Standards for Qualitative Research (JARS-Qual) [52]. Demographic data are presented in Table 1. Quantitative data and R codes are presented in the Supporting files. Narrative data (transcripts) cannot be provided because they include information that can potentially compromise the participants' anonymity. Quantitative data were analyzed using R Version 4.3.1 [53] and packages ggplot2 Version 3.5.1 [54], ggpubr Version 0.6.0 [55], readxl [56], tidyverse Version 2.0.0 [57], lubridate Version 1.9.3 [58], lavaan [59], semPlot [60], moments Version 0.14.1 [61], corrplot Version 0.92 [62], modelr Version 0.1.11 [63], psych Version 2.4.3 [64], psychometric Version 2.4 [65], glmnet Version 4.1-8 [66, 67], logistf Version 1.26.0 [68], and Caret [69]. Qualitative data were analyzed using NVivo Version 14 [70] and line-by-line narrative analysis.

### 2.2. Participants

Inclusion criteria for interviewees encompassed pain severity and duration, language requirement, and physical competence. To be more specific, each participant had experienced severe chronic pain (numerical rating scale ≧ 7 out of 10) for more than three months, could fluently communicate in either Chinese or Taiwanese, and could participate in a 90-min in-person interview. Individuals who did not meet the inclusion criteria were excluded from recruitment. Affiliation to the Daoist religion was not a recruitment criterion because, although some individuals may claim to be atheists, they actually identify with a certain religion. For example, a few participants initially claimed to be atheists but later described in the interview that they would sometimes voluntarily go to a Daoist temple or pray to a Dao-affiliated deity. In cases like this, they were classified as Dao believers. Therefore, determining whether a participant was a Dao believer was verified through two steps. First, we directly asked participants, “Which religion do you identify with?” Second, their true religious affiliation was inferred based on their responses throughout the interview.

Gender differences in religious engagement have been observed [71, 72]. Therefore, the study first interviewed three participants from each gender and continued to interview the participants until both the interviewer and notetaker agreed that the data had reached saturation [73]. In this study, we decided that the qualitative data had reached saturation when new themes stopped to emerge after three consecutive interviews (Table 2). Another halting criterion was pragmatic concerns. When the interval between the two interviews was longer than 1 month, and this occurred twice owing to the difficulty in recruiting additional participants, the research team decided to halt the recruitment of participants.

### 2.3. Questionnaires

To quantitatively measure the level of resilience from pain, the PRS was adopted [8]. It is a 14-item scale for the assessment of the cognitive positivity and behavioral perseverance when facing unbearable pain. In a previous study with a sample of patients with chronic pain, scoring lower on the PRS was found to indicate a higher risk of opioid use and unemployment due to pain [74]. Ninety-six individuals (mean age = 40.52, SD = 11.07) with chronic pain were recruited for a preliminary investigation of the internal consistency and construct validity of the Mandarin translation of the PRS for this study. This preliminary investigation showed good internal consistency for the translated scale (Cronbach's alpha = 0.96). The translated PRS showed a positive correlation with the quality of life (measured using the World Health Organization [WHO] Quality of Life Brief Version [75]) (*r* = 0.55, *p* < 0.001) and a negative correlation with depressive symptoms (measured using the Patient Health Questionnaire-9 [76]) (*r* = −0.40, *p* < 0.001). The sample mean score was 33.05 (SD = 14.16), and the median was 32. The Shapiro–Wilk test revealed no significant skewness of the score distribution (*p* = 0.052).

### 2.4. Plans for Qualitative Data Collection and Analysis

The planning of the interview questionnaire and analysis of the narrative data followed the narrative-analysis approach [77–79]. The participants' subjectivity was emphasized throughout the study. As part of the mixed-method study, this step helped researchers gain insights into the underexplored aspects of the research topic. Open-ended interview questions were derived from the literature review and designed to understand the individuals' perceptions of the past, present, and future when dealing with chronic pain in this cultural context. The preplanned questions encompassed topics such as the experience of pain, pharmacological and nonpharmacological treatment experiences, interpersonal relationships, personal interpretation of the pain experience, and aspirations for the future. One researcher used the semistructured questionnaire to conduct the interviews, while the other researcher maintained notes on the participants' nonverbal behaviors during the interviews. Both interviewers participated in debriefing sessions after each interview. Observations of how each interview unfolded, participants' nonverbal behaviors, researchers' emotions during and after each session, and other reflections on the interview were documented. Each transcript was reviewed and analyzed approximately 2 weeks after every interview.

Spontaneous Suicide Attempt References: No questions directly referenced suicide attempts throughout the interview. Data included spontaneous referencing of suicide experience or suicide attempts through the open-ended questions. For the purposes of this study, a suicide attempt is defined as behaviors led by the ideation to end one's life [80].

Thematic analysis was employed to identify patterns and themes within the qualitative data, following the six-phase framework outlined by Braun and Clarke [81], aided by NVivo 14 to systematically code and analyze the transcripts. Once the interview transcripts were imported into NVivo, data were organized into folders corresponding to participant characteristics. Transcripts were repeatedly read by the research team, and added annotations as a note-keeping method. Open coding was conducted using NVivo's code feature. In the process of line-by-line coding, new codes were created when existed codes could not delineate the emerging phenomenon. Saturation table was created with NVivo to demonstrate when each theme emerged and when new theme stopped to emerge. With the inductive approach, themes emerged from the collection of codes. Initial themes were reviewed repeatedly to ensure internal consistency and distinctiveness. Coded data extracts were reexamined for coherence, and themes were refined during the process. In the step of writing up report, we adopted the query tools in NVivo to facilitate the extraction of relevant coded segments.

### 2.5. Plans for Statistical Analysis

This step of the study allowed us to triangulate the findings from our qualitative component with established research on the prediction of suicide attempts. Similar to previous studies on factors related to suicide attempts [34], this study also applied logistic regression as the primary statistical analysis method. The role of pain resilience and gender in predicting the probability of a participant attempting suicide was investigated using a logistic regression on whether a participant made suicide attempts. To reduce the bias caused by the fairly small sample size, the values of the coefficients in the logistic regression were estimated using penalized maximum likelihood [82]. References to suicide attempts were coded dichotomously (0 = no, 1 = yes). According to the mean and standard deviation of the PRS derived from our preliminary investigation of the translated PRS, the total score on the PRS was categorized into very low (≦ 19), moderate (20–32), and high (> 33). The predictive variables of the logistic regression on the probability of suicide attempts were dummy gender variables and levels of PRS.

(1) $$\begin{array}{c} S\left(u_{i}\right)=\alpha +\beta G_{i}+\gamma_{1}P_{1}+\gamma_{2}P_{2}+\varepsilon_{i}. \end{array}$$

*S*(*u*_*i*_) = logit(*u*_*i*_)* = *log(*u*_*i*_/(1 − *u*_*i*_)), where *u* is the probability of attempting suicide, *G*_*i*_ is the dummy regressor for gender (1 for men; 0 for women), and *P*_*i*_ is the dummy regressor for levels of pain resilience, indicated in Table 3.

## 3. Results

**Figure 1:**
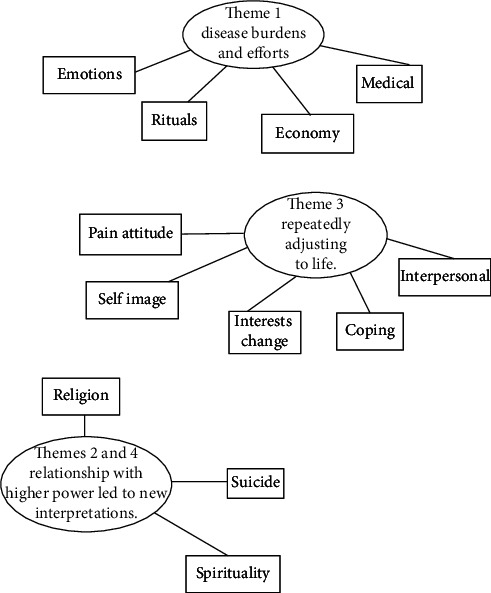
Final theme map derived from the interviews.

Initially, 24 individuals with chronic low back pain were interviewed. Among them, 7 participants identified as atheists, one as Catholic, two as Christians, two as Buddhists, and 12 as Daoists. However, two of the seven individuals, participants CML03 and CHY05, were classified as Daoists because they expressed disappointment toward their religion in the interview (see Table 1 for participant demographics). Quotes are provided in the following sections. Only transcripts from those individuals who identified as belonging to the Dao religion were further analyzed. Line-by-line analysis of the transcripts identified the religious practices that participants have followed to alleviate pain and the following emotional experiences that resulted in changes in their spirituality level. Initial themes were presented in Supporting file 1. The final themes were presented in Figure 1. Theme 1 revealed the disease burden of chronic pain and the efforts made to manage that pain. Some participants asked for guidance from the deities before making important medical decisions, while others engaged in other religious activities when in pain.

Participants shared details regarding their religious practices as follows:

“My mother went to temples to draw fortune sticks before my first back surgery.” (WBS02)

“There is a famous Guan Sheng Di Jun (關聖帝君) temple in my hometown. I went to ask for guidance from the deity there before my back surgery… In retrospect, I think that perhaps a higher force led me to the doctor who performed the surgery.” (CJC20)

“I read the *Dì zàng* (地藏) and *Guan Yin* (觀音) scriptures, although I do not understand them… I think that pain is partly psychological… I feel that reading the scriptures helps me calm down.” (CCL06)

“I would pray at home and then visit a Guan Yin (觀音) temple… I used to pray to Mazu (媽祖), and I tried to join the annual pilgrimage of Mazu (媽祖). However, it was too stressful for my body… I mostly pray for inner peace.” (CWH18)

“I would go to the Mazu (媽祖) temple near my house. I prayed for better health and even asked for the deities' guidance on the surgery by drawing a fortune stick.” (HJL16)

“At first, I did not know what was causing this immense pain; thus, I went to the temple to ask the deities.” (CHC14)

“When I do not understand something, I ask the deities… I burn incense and pray so that my pain gets alleviated… I think that I am seeking a sense of security.” (CTS22)

It can be observed that most participants wished to receive some form of protection or assistance from their deities. In the traditional Dao belief system, believers are considered servants of the deities. In the Dao religion, the gesture of praying not only includes an internal dialog with the deities but also incorporates ritual behaviors and objects that embody the abstract concept of worshiping the deities. Dao rituals typically include prayers along with incense, food, paper money, or other offerings, with worshipers hoping to exchange them for protection, express gratitude, or convey other wishes [83]. Smoke from burning incense is believed to purify the surrounding atmosphere and serve as a medium to deliver messages to the deities. Food and other offerings are considered as tokens of gratitude to the deities [84].

When their wishes are not granted, believers are likely to think that the offerings were inadequate; therefore, some donate more money to the temple to perform a larger-scale ritual, hoping to change their destiny, such as participants LDW17, CML03, and SMF12. Theme 2 revealed their relationship with the higher power. The spiritual element in these prayers and rituals lies in not only the connection with the deities but also the reflection on one's actions. Has not enough been done to please the deities? Is there something that one has failed to do to escape the punishment? What are the lessons from Heaven? Is one's faith in one's religion insufficient? This type of questioning allows for inner self-dialog and provides an opportunity for self-reflection.

Subsequently, a pattern of negative emotions emerged after endless efforts in religious practices when all other attempts had failed. This led to repetitive suicidal behaviors aiming to free oneself from the pain. Negative emotions among the participants commonly manifested as accusations leveled against their fate or religion.

“It is pointless… We visited so many temples… every time someone said that a temple was effective, we would go there. Did it work? We have lost several lawsuits, and the pain persists. I have no faith in religion anymore.” (SMF12)

“I used to believe that the deities would help me find a good job and a nice husband… I tried fortune telling, and I drew fortune sticks. Now, I have no faith left… Why are the deities giving me so much pain in my marriage and my body?” (CML03)

“When I was a child, I believed that praying induced a sense of calmness in me. Gradually, I have stopped believing in any religion. It is ineffective. I prayed for better height, good grades, and for the pain to disappear. Nothing worked… What have I done to deserve this? Why do I have to suffer like this?” (CHY05)

“Psychics told me that my pain was caused by enemies from a past life… The temple asked for more money to solve the issue, and thus we gave it to them… I do not even know who my enemies are; how do I believe in what they said? Besides, I am still experiencing immense pain every day.” (LDW17)

“I often complained to the deities… this pain is interrupting my life… and the tendon point injections are extremely painful…” (HPW24)

After repeatedly putting effort into medical care, pleasing gods, and facing disappointments, the participants progressed to exhaustion [85]. Although these thoughts were presented as accusations against a higher power, they reflected helplessness and exhaustion after an arduous journey of negotiations and efforts. This struggle to make sense of the source of suffering links the individual to the context in which they live [86]. Theme 3 revealed the various aspects of life that the participants were repeatedly forced to make adjustments to. Most participants had spent years trying various methods to treat their chronic pain; however, their efforts were in vain. In some cases, the pain had exacerbated over the years. The underlying assumption of these accusations against faith is that an omniscient higher power exists that dictates the occurrence of disease. Although it resembles the anger stage mentioned in Kübler-Ross's model of five stages of grief [87, 88], anger is not the main or sole theme in this phase. Feelings of disappointment are also present. Disappointment can occur when one's wishes are not granted [89]. However, when the wish to alleviate the pain remains unrealized, some abandoned their faith. Without expectations, the risk of facing disappointments diminishes [90, 91].

**Figure 2:**
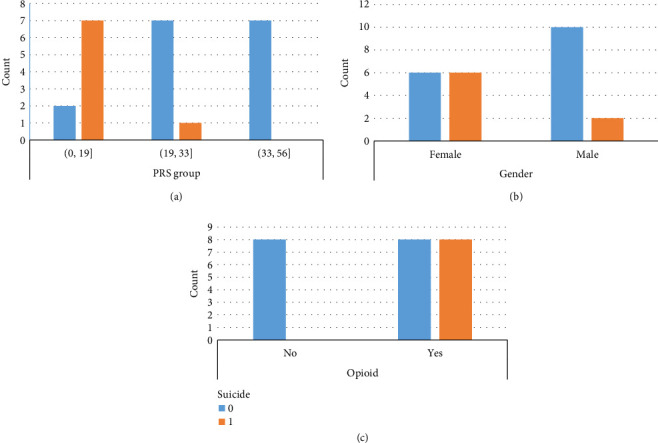
Frequency of references to suicide attempts by participant characteristics PRS group indicates the grouping according to scores rated on the pain resilience scale. Suicide = 0 indicates no suicide attempt; Suicide = 1 indicates with suicide attempt. Opioid = 0 indicates no use of opioid for pain control; and Opioid = 1 indicates the use of prescription opioid as a method of pain control.

Accusing a higher power may be a mere prelude to more extreme ways to untie oneself from the ordeal. The frequencies with which individuals made spontaneous references to suicide attempts were characterized by gender and the PRS score (Figure 2). Most participants who rated very low on the PRS had experience of attempting suicide, whereas no participant rated high on the PRS had such experience.

It is difficult to determine the meaning of suicidal behaviors among individuals with pain because the cause is heterogeneous [92, 93]. Often, when suicidal ideation was brought up in a dialogue, the individuals implied that they wanted a better life without pain or judgments. Among the many attempts at untying oneself from pain, suicide was one option [94]. To paraphrase, committing suicide was not the intention but rather a means to obtain a better quality of life or live a life without judgments. At last, Theme 4 emerges. It appeared to be the same component as Theme 2 but from a different perspective.

“My parents told me to endure the pain and overcome it… They did not listen to me. Finally, I gained their attention after my seventh or eighth suicide attempt.” (WBS02)

“I frequently cut my wrist and tried overdosing on medications… I then realized that the world would not change for me… my parents would continue to abuse me… The only way to free myself was to not care about these people… I then stopped hurting myself.” (CML03)

“I tried committing suicide. I took a considerable amount of zolpidem and morphine tablets and then drove onto the highway… I believed that I would not feel the pain anymore… I saw myself being resuscitated. I think that Pusa (菩薩) saved me. My fever went on and off for five days, and then I woke up. It must be Pusa's (菩薩) doing.” (CHC14)

“I had plans and thoughts about how to do it… and then I went to the temple to pray. I believe in Mazu (媽祖). When I was praying, it occurred to me that my child would not have wanted this. I have certain responsibilities to my child. From then on, that is the only voice in my head.” (CTS22)

How the participants approached discussions about suicide revealed the variety in their outlook on religiosity. For some, it signified the disconnection of oneself from others, whereas for others, it resembled a fresh start. Some participants have found their way back to religion.

The results of the logistic regression on the probability of making suicide attempts revealed that, compared with participants who rated very low on the PRS, the likelihood of individuals with moderate PRS scores attempting suicide decreased by 11.60 (= 1/exp(−2.45)) times, and the likelihood further decreased by 38.70 (= 1/exp(−3.66)) times in individuals with high PRS scores (Table 4).

Recovering from a suicide attempt paved the way to the meaning-making process. Religiosity was presented in individuals who were on the verge of suicidal behaviors and in those who had attempted suicide. Most individuals tried to make sense of the suicide experience and attached a spiritual interpretation to it.

## 4. Discussion

This study illustrates how pain resilience and Dao religious practices shape the behaviors and thoughts of individuals experiencing pain. Using a mixed-method study, the roles of resilience and religion in mitigating an adverse pain outcome, suicide attempts, were explored through qualitative study and then verified with statistical analysis. Consistent with previous studies, we found that a higher level of resilience was associated with reduced odds of suicidality in individuals with pain [95] and that spiritual coping was related to remission from suicide attempts [33]. Moreover, we investigated how Dao practice as a religious resource was adopted in pain management and subsequently assisted with recovery from suicide attempts. Unlike previous study, we did not find female gender to be at a higher risk of suicide attempts. This will be discussed in a later paragraph.

In Dao religion, deities are considered powerful ancestors who can perform miracles [84]. Pain is beyond one's control; however, the common belief is that the deities are in charge of everything. The social power of religion is presented through interactions between worshipers and religious figures [96]. Some individuals asked for mercy, others sought answers, and some sought support from their religion. As presented in Theme 2, participants performed various religious practices or rituals, expecting to receive help from the deities to manage their problems in exchange [97]. Praying was the most common practice among the participants. Other rituals, such as fortune telling, drinking spell water, rituals for changing luck, drawing fortune sticks, or burning paper money, were also performed. Spirituality was manifested through connections with the deities and self. It was cultivated through ritual practices, and how these practices influenced the participants' interpretation of the pain experience. The transformation from Theme 2 to Theme 4 presented the course of spiritual discontent to spiritual reconnection. It indicated a transformation from a negative religious/spiritual coping to a positive one [28]. Also, the participants tried to reappraise their experience from another perspective. This meaning-making process was a presentation of healthy spirituality, which may contribute to the strengthening of resilience [12, 97]. Although it did not always appear to be a pleasant experience for the participants, they managed to see that connections existed between the deities and the self.

Even though religiousness has been found to positively impact mental health [97], the findings of the present study indicated that a risk of helplessness emerged when the participants' wishes were not granted after extensive religious practices. In a review study on the regret and disappointment, it was found that people tended to “try harder” in order to reach a desired outcome [89]. In our case, participants prayed more intensely, paid more for rituals, or visited more temples in the hope of a better pain control. Generally, investing efforts increases the probability of a more desirable outcome. However, disappointment arises when the outcome is not as good as expected in an uncontrollable situation. A previous study reported that individuals with religious beliefs tended to have more severe depression [98]. The interviews revealed that participants had several unfulfilled wishes.

Understanding the risk factors associated with suicide in individuals with chronic pain is important before approaching this issue in a clinical context [33, 80, 94]. When learning about suicidal ideation, clinicians are trained to either respond or intervene. The findings of the present study, similar to those of previous studies [29, 99, 100], demonstrate that religion can be an important resilience resource against suicidality among individuals with pain.

Scholars have linked the suicidality in chronic pain to meaning in life [101]. A similar finding can be noted in the realizations presented by the participants in this study. When the participants expressed the idea of suicide, it connoted a series of searches for meaning in their lives [102]. This often involved reflecting on one's life, condition, and relationships with others. When communicating with the deities, questioning one's faith, having doubts, or asking questions to a higher power can be a stage of meaning integration. First, it may present itself as a way to discontinue life when a participant tries to end the suffering by committing suicide. The turning point is often the realization of a connection between oneself and the higher power or between oneself and loved ones. This gain in spirituality confers the strengthening of resilience to adversity [12]. The integration of life purposes after surviving a significant suicidal incident was noted.

Prior to this study, few studies had examined how pain resilience and Dao religion were associated with the suicidality of chronic pain. However, this study has limitations. First, no quantifiable scale was used in the study to determine whether an individual was spiritually healthy. This could be helpful in understanding the spiritual well-being of each participant. Second, the data collection may have been biased because only participants with chronic pain were recruited. Only individuals in pain would continue to seek medical help from hospitals. Narratives of individuals who had recovered from the pain were not included. The third limitation is the very small sample size in our qualitative study, which limited the power of the statistical analysis. Inconsistent with previous study, our logistic regression on suicide attempts did not find female to be at a higher risk of suicide attempt [34]. There are many reasons for this inconsistency. For example, in a study that included 210 women and 190 men, women showed better adaptation to pain despite higher levels of pain anxiety and pain intensity. In contrast, fear-avoidant behavior, a passive coping strategy, was found only to be associated with pain intensity in men [103]. Coping and adjustments to pain may also result in different pain outcomes. However, we would still like to emphasize that although past research has found gender to play a role in both suicide attempts and resilience, inferences drawn from the results obtained from a limited sample size should be interpreted with caution. Nevertheless, we hope that this study can provide a deeper understanding of how the less investigated Dao religion plays a role in the suicidality of chronic pain. Future studies may consider including a quantifiable measure that can help assess participants' spiritual health. Additionally, when sufficient resources allow for multiple recruitment methods, future studies should attempt to recruit individuals who have recovered from chronic pain.

## Figures and Tables

**Table 1:** Demographic details of the participants associated with Dao religion.

Gender	Participant pseudonym	Age (in years)	Education level	Years in pain	Frequency of religious practices
Male	WBS02	38	College	10	Daily
	CHY05	27	High school	12	Irregular
	CCL06	63	8th grade	12	Daily
	CCK07	40	College	6	On festivals
	HJL16	49	9th grade	13	Monthly
	LDW17	45	9th grade	5	Daily
	CSC19	38	High school	6	Daily
	CJC20	44	9th grade	19	On festivals

Female	CML03	43	9th grade	7	Irregular
	SMF12	47	High school	11	On festivals
	CHC14	52	High school	3	Irregular
	CWH18	42	High school	18	Daily
	CTS22	33	College	2	Daily
	HPW24	40	College	20	On festivals

Average		42.93 ± 8.29		10.29 ± 5.61	

**Table 2:** Saturation table showing theme emergence across interviews.

Interview	1	2	3	4	5	6	7	8	9	10	11	12	13	14
Medical	X	X	X	X	X	X	X	X	X	X	X	X	X	X
Religion	X	X	X	X	X	X	X	X	X	X	X	X	X	X
Rituals	X		X	X	X	X	X	X	X	X	X	X	X	X
Emotions	X		X			X	X	X	X	X	X		X	X
Economy	X		X	X	X	X	X	X	X	X	X		X	X
Coping		X	X		X		X				X	X	X	X
Pain attitude		X	X							X	X			X
Suicide			X	X		X	X		X		X	X		
Interpersonal				X	X		X	X	X	X	X	X	X	X
Self-image						X					X			
Spirituality							X	X	X	X	X	X		X
Interests change											X	X		
New theme	5	2	1	1	0	1	1	0	0	0	1	0	0	0

**Table 3:** Dummy regressor coding scheme for the three levels of pain resilience in the regression equation.

Levels of pain resilience	*P*_1_	*P*_2_
Very low (≦ 19)	0	0
Moderate (20–32)	1	0
High (> 33)	0	1

**Table 4:** Logistic regression on the probability of attempting suicide (with penalized maximum likelihood estimates).

Coefficients	95% CI	Odds	*z*-value	*p*
Intercept	(−0.02; 3.87)	4.81	1.57	0.052
Gender_male	(−3.86; 0.92)	0.28	−1.27	0.253
PRS (19, 33]	(−5.14; −0.36)	0.09	−2.45	0.020
PRS (33, 56]	(−8.70; −1.05)	0.03	−3.66	0.003

## Data Availability

Demographic data are presented in the manuscript. Narrative data (transcripts) cannot be provided because they include information that can potentially make the participants identifiable. R codes and raw data for statistical analysis are provided as Supporting files. Preliminary analysis of the qualitative data are available in the Supporting information of this article.

## Supporting Information

Supporting Information Supporting 1: Excel file (*n* = 96), PRS scores, is the raw data for the preliminary of Mandarin Chinese version of PRS. Related information is mentioned in the Questionnaires section under Methods.

Supporting Information Supporting 2: Excel file, PRS and demo test, is the raw data for the logistic regression on the probability of attempting suicide.

Supporting Information Supporting 3: Text file, PRS 1to 14_ *n* = 96, is the R code for the preliminary investigation of the internal consistency and construct validity of the Mandarin translation of the PRS. Related information is mentioned in the Questionnaires section under Methods.

Supporting Information Supporting 4: Text file, test with penalized ML, is the R code for the logistic regression on the probability of attempting suicide. Related results are displayed in Table 4 and Figure 2.

Supporting Information Supporting 5: Docx file, initial theme map derived from the interview.
