# Supplementary material for: Do We Ask What the Deities Can Do for Us? The Roles of Dao Religion and Resilience in Suicidality in Chronic Pain
Source: Pain Res Manag. 2025 Apr 17;2025:3056383. doi: 10.1155/prm/3056383 (PMC12021491; doi:10.1155/prm/3056383)
Supplement: Supporting Information — Supporting 5: Docx file, initial theme map derived from the interview. [file 3056383.f5.docx]

**Theme 2**

**Relationship with higher plays a role.**

**Theme 4**

**New interpretations**

**Theme 3**

**Adjustments to life.**

Medical history

**Theme 1**

**Disease Burdens**

Pain attitude

guilt

Interests

change

Body sensation

suicide

passive

active

self

others

nature

higher

changes

change

healthcare

work

family

changes

negative

positive

spirituality

emotions

interpersonal

aspiration

religion

coping

economy

Self image
